# Comprehensive Extraction and Biological Activities of Mycosporine-like Amino Acids and Glyceroglycolipids Extracts from Two Macroalgae *Ecklonia kurome* and *Ulva lactuca*

**DOI:** 10.3390/foods14030440

**Published:** 2025-01-29

**Authors:** Xin Wei, Xiaoqi Hu, Tianhuan Li, Yuxiang Li, You Yu, Xiujing Jiang, Haonan Wang, Jie Yang, Xue Jiao, Xinghu Zhou, Yingying Sun

**Affiliations:** 1 Jiangsu Key Laboratory of Marine Bioresources and Environment, Jiangsu Ocean University, Lianyungang 222005, China; weixin@jou.edu.cn (X.W.); 2022220838@jou.edu.cn (T.L.); yangjie@jou.edu.cn (J.Y.); 2 Co-Innovation Center of Jiangsu Marine Bio-Industry Technology, Lianyungang 222005, China; 3 Jiangsu Institute of Marine Resources Development, Lianyungang 222005, China; 4 Jiangsu Coast Development Group Co., Ltd., Nanjing 210095, China; jiaoxue92@163.com (X.J.); zhouxinghu@jsyhkf.com (X.Z.)

**Keywords:** mycosporine-like amino acids, glyceroglycolipids, marine macroalgae, *Ecklonia kurome*, *Ulva lactuca*, antioxidant, moisturizing activities

## Abstract

Mycosporine-like amino acids (MAAs) and glyceroglycolipids have promising applications in various fields, but limited research exists on their simultaneous extraction from macroalgae. This study optimized the key parameters (liquid–solid ratio, extraction temperature and extraction time) in the extraction of MAAs and glyceroglycolipids from *Ecklonia kurome* and *Ulva lactuca* using single factor and response surface experiments. The yields of MAAs from *E. kurome* and *U. lactuca* were 169.71 mg/g and 177.33 mg/g, respectively, while glyceroglycolipids were extracted from the residue with yields of 163.51 mg/g and 213.45 mg/g, respectively. Both extracts showed concentration-dependent antioxidant activities, with the MAAs extracted from *U. lactuca* demonstrating the strongest effect. The addition of MAA extract to flaxseed oil significantly reduced oxidation rancidity, highlighting its potential as a natural antioxidant for oils. The glyceroglycolipid extract from *E. kurome* exhibited significant moisture absorption, and a water-retaining agent prepared from this extract showed excellent moisture retention and resistance to high temperatures, freezing, and pressure. A silica gel column chromatography method confirmed the presence of MGDG in the purified glyceroglycolipid extract. These findings suggested that *E. kurome* and *U. lactuca* can be converted into high-value-added compounds with potential applications in food, cosmetics, and pharmaceuticals.

## 1. Introduction

Mycosporine-like amino acids (MAAs) are natural secondary compounds widely distributed among a wide range of aquatic organisms, especially cyanobacteria and macroalgae [1,2,3]. They are water-soluble, composed of a core cyclohexenone or cyclohexenimine ring, and have low molecular weights (<400 Da) [4]. Due to their conjugated double bonds, they strongly absorb light between 310 nm and 360 nm [5], which gives them application potential as sunscreens. Among the most representative products, shinorine, porphyra-334, and palythine have been isolated and purified from macroalgae [6]. Recently, MAAs have garnered increasing attention due to their promising effects on antioxidation, anti-inflammation, anti-aging, and DNA protection [7,8,9].

Glyceroglycolipids are sugar compounds composed of hydrophilic glycosides and lipophilic glycerol groups connected by glycosidic bonds [10], commonly found in the thylakoids of plants [11]. In the last few decades, many marine macroalgae, such as *Ahnfeltia tobuchiensis* [12], *Chondria armata* [13], *Gracilaria verrucosa* [14], and other Rhodophytes species [15,16]; *Fucus vesiculosus* [17], *Laminaria gurjanovae* [18], *Sargassum wightii* [19], and other Phaeophytes species [20,21,22]; *Codium tomentosum* [23], *Ulva rigida* [24], *Ulva pertusa* [25], *Tydemania expeditionis* [26], and other Chlorophytes species [27,28,29], have been shown to contain various types of glycoglycerolipids. Recently, glyceroglycolipids have been divided into three types according to their glycosyl and acyl structures: monogalactosyl–diacylglycerol (MGDG), digalactosyl–diacylglycerol (DGDG), and sulfoquinovosyl–diacylglycerol (SQDG) [13,30]. Due to their unique amphiphilic structure, glyceroglycolipids possess various significant biological properties such as anti-tumor [31], antioxidant [32], antiviral [33], anti-inflammatory [34], and antibacterial [35] properties. Glyceroglycolipids can be used as food additives and moisturizing agents and can also be further applied in the pharmaceutical industry to develop anti-tumor, antiviral, and other drugs.

Previous studies have investigated the extraction of MAAs and glyceroglycolipids separately from various species of macroalgae, with significant variations in yield, bioactivity, and chemical composition depending on the species and extraction methods employed. For example, MAAs from red algae such as *Porphyra* sp. have been widely studied for their sunscreen properties, while the antioxidant potential of glyceroglycolipids has been highlighted in brown and green algae. Currently, marine algae are being extensively studied as sources of food, medicine, cosmetics, fertilizers, and bio-energy [36]. Undoubtedly, natural active ingredients from marine algae are expected to replace chemical products in the near future. As mentioned above, MAAs and glyceroglycolipids have application potential in various fields such as biology, medicine, food, cosmetics, and the environment. However, most researchers have focused on the extraction of a single compound and have similar extraction methods. Studies on the simultaneous separation and purification of MAAs and glyceroglycolipids are relatively rare, largely due to the low abundance and complex separation challenges of these compounds [10]. Furthermore, the economic feasibility and scalability of such extraction methods have not been adequately addressed. *E. kurome* (named by J.W. Hornemann, 1828) is an economically large brown alga that is widely cultivated in northwest Asia, with both its yield and commercialization level leading the industry. *U. lactuca* (named by Linnaeus, 1753), belonging to Chlorophyta, is primarily distributed along the coastal regions of China. These two species of macroalgae are rich in essential nutrients such as polysaccharides, proteins, and vitamins. More importantly, they are rich in amino acids and glycolipids. However, their further development and utilization, particularly in high-value bioactive compounds, remain underexplored. In this study, MAAs and glyceroglycolipids were simultaneously separated from *E. kurome* and *U. lactuca* due to the water solubility of MAAs and the lipid solubility of glyceroglycolipids. Subsequently, the antioxidant and moisturizing properties of these extracts were evaluated, especially the antioxidant role of these extracts in flaxseed oil. This is the first instance of separating MAAs and glyceroglycolipids simultaneously from macroalgae, which will promote the further utilization of marine algae resources in food industry.

## 2. Materials and Methods

### 2.1. Macroalgae Materials and Reagents

*E. kurome* and *U. lactuca* were collected from the coastal waters near Weihai, Shandong Province (longitude 122°11′E, latitude 37°33′N), at a temperature of 26 °C and a relative humidity of 65%. Macroalgae were rinsed in distilled water, drained on paper towels, and dried in an oven at 40 °C for 12–24 h. The dried macroalgae material was then cut into small pieces and ground into powder using a blender. The powder was stored at −20 °C until extraction. MGDG standards were purchased from Avant Polar Lipids (Alabaster, AL, USA). The acetonitrile (chromatography-grade) used for high-performance liquid chromatography (HPLC) was purchased from Sigma Aldrich (Shanghai, China) Trading Co., Ltd. 2,4,6-Tripyridyl-s-triazine (TPTZ), sodium thiosulfate, thiobarbituric acid (TBA), potassium ferricyanide, potassium bromide (KBr) and other reagents or compounds were of analytical grade and obtained from Sinopharm Chemical Reagent Co., Ltd., in Shanghai, China.

### 2.2. Extraction of Crude MAAs and Glyceroglycolipids from Macroalgae

#### 2.2.1. Optimization of MAA Extraction Conditions

Single factor experiments and Box–Behnken central composite experiments were conducted to optimize the key parameters involved in MAA and glyceroglycolipid extraction. The detailed technical route is shown in the Figure S1. Firstly, MAAs were extracted from dried macroalgae powder. Five grams of macroalgae powder was weighed and mixed with a 25% methanol solution as per the proportions outlined in Table S1. The mixture was oscillated in a water bath at 180 rpm using a constant rate oscillator. The extraction temperature and time parameters are detailed in Table S1. After extraction, the solution was allowed to stand for approximately 30 min before decanting. This process was repeated, and the combined extract was collected. The supernatant was obtained through centrifugation and subsequently evaporated (Roto evaporator purchased from Zhengzhou Brilliance Instrument Co., Ltd., Zhengzhou, China) under reduced pressure. Anhydrous ethanol was added to the concentrated extract at a ratio of 1:4 and stored at −20 °C for 6 h to remove polysaccharides. The concentrated MAA solution was obtained after further evaporation under reduced pressure and finally freeze-dried to produce crude MAA extracts from macroalgae. The yield of MAA extracts from *E. kurome* and *U. lactuca* was calculated using the following formula, where M_1_ was the quantity of crude extracts (mg), and M_2_ was the quantity of macroalgae powder (g).

Based on the results of the single factor tests, a three-factor and three-level experimental design was conducted using the Box–Behnken central composite design for response surface experiments. The extraction conditions for MAAs were optimized with liquid–solid ratio (A), extraction temperature (B), and extraction time (C) as the key optimization parameters, with MAA extract yield (mg/g) as the response value across 17 experimental runs. Detailed factor values and levels for the response surface tests are provided in Table S2. Design-Expert software version 13.0 was utilized for the experimental design, regression analysis, and determination of optimal extraction processes.

Yield (mg/g) = M_1_/M_2_ (1)

#### 2.2.2. Optimization of Glyceroglycolipid Extraction Conditions

The remaining residue from MAA extraction was dried and 5 g of powder was precisely weighed to prepare glyceroglycolipids. Methanol aqueous solution was added as per the amounts and ratios shown in Table S3. The mixture underwent extraction in a water bath at 180 rpm with constant temperature oscillation. The extraction temperature and time were optimized as per Table S3. After resting for about 30 min, the same extraction process was repeated before merging the extracts. The crude glyceroglycolipid extract was obtained through filtration using a Brinell funnel, concentrated under reduced pressure at 45 °C, and finally freeze-dried. Similarly, response surface methodology was applied to optimize the extraction conditions of glyceroglycolipids based on single factor experiments and Box–Behnken central composite design. A four-factor and three-level experiment was designed with the liquid–solid ratio (A), extraction temperature (B), methanol ratio (C), and extraction time (D) as response factors and the yield of glyceroglycolipid extract (mg/g) as the response value. Detailed factor values and levels for the response surface tests are provided in Table S4. The yield of glyceroglycolipid extracts from *E. kurome* and *U. lactuca* was calculated using the above formula.

### 2.3. Assessment of Antioxidant Activity of Different Extracts

#### 2.3.1. Determination of DPPH Free Radical Scavenging Ability

To 100 µL of 0.2 mmol/L DPPH free radical solution, 100 µL of MAA or glyceroglycolipid extracts at different concentrations was added and mixed thoroughly. The mixture was allowed to stand at room temperature for 30 min, and the absorbance (OD) was measured at 517 nm (spectrophotometer purchased from Shanghai Youke Instrument Co., Ltd., Shanghai, China). Each experiment was conducted in triplicate. The DPPH free radical scavenging capacity was calculated using the following formula, where A_1_ was the OD of the sample with DPPH, A_2_ was the OD of the sample without DPPH, and A_0_ was the OD of the control without sample. The IC_50_ value, representing the concentration of MAA or glyceroglycolipid extract required to scavenge 50% of DPPH free radicals, was also determined.

DPPH free radical scavenging rate (%) = [1 − (A_1_ − A_2_)/A_0_] × 100% (2)

#### 2.3.2. Assessment of ABTS Free Radical Scavenging Ability

A total of 0.01 g of MAAs or glyceroglycolipid extract was dissolved and filtered through a 0.45 µm membrane. Then, 100 µL of the sample was mixed with 100 µL of ABTS free radical solution and incubated at room temperature for 10 min in the dark. The absorbance at 734 nm was measured, and the experiment was repeated in triplicate. The ABTS free radical scavenging capacity was calculated using the following formula, where A_1_ was the OD of the sample with ABTS, A_2_ was the OD of the sample without ABTS, and A_0_ was the OD of the control without sample, and the IC_50_ value was determined.

ABTS free radical scavenging rate (%) = [1 − (A_1_ − A_2_)/A_0_] × 100% (3)

#### 2.3.3. Analysis of Ferric Ion Reducing Antioxidant Power (FRAP)

Solutions of MAA or glyceroglycolipid extracts with varying concentrations were prepared, and 300 µL of each sample was mixed with 2.7 mL of TPTZ solution. The reaction mixture was incubated at 37 °C for 30 min, and the absorbance at 593 nm was measured. The FRAP values were calculated using a standard curve with FeSO_4_•7H_2_O, and the experiment was repeated three times for each group.

#### 2.3.4. Determination of Total Reducing Capacity

Solutions of MAAs or glyceroglycolipid extracts at different concentrations were prepared. To each test tube, 0.1 mL of sample, 2.5 mL of PBS buffer (pH 6.6), and 1.0 mL of 1% potassium ferricyanide solution were added. The mixture was heated in a water bath at 50 °C for 20 min and then cooled rapidly with cold water. After centrifugation, 2.5 mL of the supernatant was mixed with 0.45 mL of 1% ferric chloride solution, adjusted to 5 mL with distilled water, and left at 25 °C for 10 min. The absorbance at 700 nm was measured, and the total reducing capacity was calculated by comparison with a negative control. The experiment was conducted in triplicate.

#### 2.3.5. Evaluation of the Ability of MAA Extracts to Protect Flaxseed Oil from Oxidation

Different concentrations (0.8, 1.6, 2.4, 3.2%, *v*/*v*) of MAA extracts from *U. lactuca* were added to flaxseed oil. Flaxseed oil without any antioxidant served as the negative control, while flaxseed oil supplemented with 0.2 g/kg butyl hydroxyanisole (BHA) served as the positive control. The samples were incubated at 60 °C using the Schaal oven method. The peroxide value (POV), acid value (AV), P-anisidine content, and malondialdehyde (MDA) content were measured every 48 h over a period of 14 d, and the experiment was repeated three times for each group. Standard methods were used for peroxide value (GB 5009.227-2023), acid value (GB 5009.229-2016), P-anisidine content (GB/T 24304-2009), and malondialdehyde content (GB 5009.181-2016). In the process of lipid oxidation, peroxides reacted with potassium iodide to form free iodine, which was titrated with sodium thiosulfate solution. The peroxide value was calculated by measuring the volume of sodium thiosulfate solution consumed. The acid value was calculated by titrating the free fatty acids in the oil with a standardized potassium hydroxide solution, using phenolphthalein as an indicator. The volume of potassium hydroxide consumed was used to calculate the acid value. The determination of p-anisidine content was carried out by a colorimetric method. Under certain conditions, p-anisidine reacted with aldehydes and other compounds in the oil to form a product that showed maximum absorption at a wavelength of 350 nm. The absorbance was linearly related to the p-anisidine content, allowing for its quantification. Under acidic conditions, MDA reacted with TBA to form a reddish-brown compound, 3,5,5-trimethylpyrazole-2,4-dione, which absorbed maximally at 532 nm. The absorbance showed a linear relationship with MDA content, and the MDA concentration in the oil was determined by measuring the absorbance.

### 2.4. Determination of Moisturizing Activity

#### 2.4.1. Hygroscopic and Hydrating Properties of MAA and Glyceroglycolipid Extracts

The hygroscopic properties of MAA and glyceroglycolipid extracts were studied using glycerol and hyaluronic acid (HA) as controls. Two groups of samples, each weighing 0.05 g, were prepared for testing. One set of samples was placed in a desiccator containing a saturated sodium carbonate solution to maintain a relative humidity (RH) of 43%, while the other set was placed in a desiccator with a saturated ammonium sulfate solution to maintain an RH of 81%. The ambient temperature was maintained at 25 °C, and three parallel tests were conducted for each group. The weights were recorded at 1, 3, 6, 12, 24, 36, and 48 h, respectively. The moisture absorption rate was calculated using the following formula, where W_1_ represented the initial mass of the glyceroglycolipid extract, and W_2_ represented the mass of the glyceroglycolipid extract after water absorption.

Moisture absorption rate (%) = (W_2_ − W_1_)/W_1_ × 100% (4)

Firstly, MAA extract, glyceroglycolipid extract, glycerol, and HA were dried to a constant weight. Then, 0.05 g sample was added to distilled water in a ratio of 3:1 liquid to solid and mixed. The mixture was placed in a silicone dryer and weighed at 1, 3, 6, 12, 24, 36, 48 h. The moisture retention rate was calculated according to the following formula, where m_2_ was the mass of MAA or glyceroglycolipid extract at saturation moisture absorption, and m_3_ was the mass of MAA or glyceroglycolipid extract placed in the dryer for a specified time.

Moisture retention rate (%) = m_3_/m_2_ × 100% (5)

#### 2.4.2. Preparation of Biological Water-Retaining Agent

An appropriate amount of acrylic acid was mixed with a sodium carbonate solution until a predetermined neutral state was achieved. The acrylic solution was then slowly added to the glyceroglycolipid extract and thoroughly mixed. Following this, a specific amount of N,N’-Methylenebisacrylamide (crosslinking agent) and potassium persulfate (initiator) was added to the mixture. The reaction was conducted under controlled conditions in a constant temperature water bath for 3 h. Afterward, the product was transferred to 80 °C for drying to remove excess water. Subsequently, the dried product was pulverized using a mortar. Finally, the powder of the glyceroglycolipid water-retaining agent with uniform particle size was obtained through sieving. The water absorption ratio of the prepared water-retaining agent served as the test index to analyze the influence of key factors such as the mass ratio of acrylic acid to glyceroglycolipid extract (10:1, 20:1, 30:1, 40:1, 50:1 g/g), initiator content (0.5, 0.8, 1.1, 1.4, 1.7%), crosslinking agent content (0.05, 0.1, 0.15, 0.2, 0.25%), and neutralization degree (60, 65, 70, 75, 80%) through single-factor tests. These factors were subsequently optimized using an orthogonal experiment (L_9_, 3^4^). Detailed factors and levels are shown in Table S5, and each experiment was conducted in triplicate.

#### 2.4.3. Evaluation of Water-Retaining Agent Performance

The water-retaining agent (1.0 g) prepared as described above was tested for its water absorption capacity using the method mentioned previously. Each sample was repeated 3 times. Samples were dried under consistent conditions, and the water absorption capacity was measured at hourly intervals to observe the dynamic changes in water absorption rate. The water absorption rate was calculated using the formula described above. To evaluate the temperature stability of the water-retaining agent, 10.0 g of the agent was accurately weighed, and its initial mass was recorded. It was then subjected to treatment at 60 °C, and the water absorption rate was measured hourly. For evaluating freezing stability, 5 samples of 0.1 g each were accurately weighed and placed into self-sealing bags. After freezing treatment at −20 °C, the samples were removed at specific time points (0, 24, 48, 72, 96 h), and the water absorption performance of each sample was tested. To assess pressure stability, the initial mass of 10.0 g of the water-retaining agent was determined after saturation. The agent was centrifuged at rotational speeds of 1000, 3000, and 5000 rpm for 30 min, after which the water absorption ratio was measured.

### 2.5. Purification and Identification of Glyceroglycolipids

#### 2.5.1. Purification of Glyceroglycolipids from Crude Extract

Chloroform was used to dissolve the glyceroglycolipid extract with a liquid to solid ratio of 10:1. Initially, chloroform and methanol were mixed in a volume ratio of 1:2 and thoroughly stirred. Next, a 0.9% NaCl solution was added to adjust the ionic strength and polarity of the solution. The mixed solution was stirred slowly for approximately 5 min at room temperature to ensure sufficient contact and reaction between the components. After allowing it to settle, the lower layer of the solution was collected and evaporated by rotary evaporation at 45 °C to obtain the glyceroglycolipid extract, which represented the enriched glyceroglycolipid fraction. Subsequently, the glyceroglycolipid extract was fully dissolved in a methanol solution (methanol: water = 2:1). Ethyl acetate was added in twice the volume of the methanol solution, and this step was repeated 3 to 4 times. The glyceroglycolipid solution was obtained by combining the extracts and concentrating the ethyl acetate phase using rotary evaporation at 45 °C. The extracts obtained above contained various components, including pigments, neutral lipids, and phospholipids. To separate these components based on their polarity differences, gradient elution was performed using solvents such as chloroform, acetone, and methanol. Specifically, pigment and phospholipid removal was achieved through gradient elution. For further purification, 5 mL of the glyceroglycolipid solution was loaded onto a silica gel column chromatography (4.0 × 40 cm). The elution rate was set at 1.0 bed volume /h (BV/h), sequentially using chloroform, acetone, and methanol. Each eluent was passed through the column 3 times the column volume, and the acetone phase was collected and concentrated by rotary evaporation at 45 °C. Next, 3 mL of the concentrated acetone phase was loaded onto another silica gel column chromatography (2.5 × 20 cm). Chloroform/methanol (9:1, *v*:*v*) was employed as the eluent, with an elution rate of 1.0 BV/h and eluting 3 times the column volume. Fractions of 10 mL each were collected into tubes for further testing and analysis.

#### 2.5.2. Thin Layer Chromatography Detection

A total of 0.1 g glyceroglycolipid extract was dissolved in 10 mL methanol. The sample was placed on a silica gel plate (Qingdao Ocean Chemical Co., Ltd., Qingdao, China, 100 × 200 mm) with chloroform/methanol/acetic acid (65:15:2, *v*:*v*:*v*) as the developing agent. Molish reagent (α-naphthol/sulfuric acid/ethanol/water, 10.5:6.5:40.5:4.5) was used as a color developing agent, and the color was developed after heating at 90 °C for 20 min. For the qualitative analysis of samples, the specific shift value (Rf) of chromogenic sites was determined and compared with the known literature.

#### 2.5.3. Infrared Spectrum and HPLC Detection

The glyceroglycolipid extract sample was mixed with KBr powder at 1:100 and ground for 5~15 min. It was then pressed into a round tablet in a paper press (pressure is usually 8000~15,000 kg/cm^2^). The KBr blank tablet was used as reference ratio for scanning, with a scanning range of 400~4000 cm^−1^ and resolution of 4 cm^−1^. The C18 column (Waters Symmetry, 4.6 mm × 250 mm × 5 μm) was used for HPLC analysis using Agilent 1260. The column temperature was set at 25 °C and the sample size was 10 µL. The mobile phase was pure water/acetonitrile (1:9, *v*:*v*), the flow rate was 0.8 mL/min, and the detection wavelength was 210 nm.

### 2.6. Statistical Analysis

Unless otherwise specified, each experiment was set up with 3 parallel samples. The data obtained were expressed as the mean ± SD. Origin 2021 and SPSS 14.0 software were used for data analysis and chart plotting. Design-Expert 13.1 software was utilized for response surface experiment design and data analysis. The Box–Behnken data were analyzed using multiple regression in Design-Expert 13.1 to determine the relationship between the independent variables and the dependent response. The results were fitted to a second-order polynomial model, which was selected based on the goodness-of-fit metrics, such as R^2^, adjusted R^2^ (R_Adj_^2^), and predicted R^2^ (R_Pred_^2^), to ensure the model’s reliability. Analysis of variance (ANOVA) was conducted with a confidence level of 95% to evaluate the significance of the regression coefficients and the model’s adequacy, and post hoc tests were conducted using Tukey’s test. A *p*-value of less than 0.05 was considered statistically significant. The normality and homogeneity of variance were judged by residual analysis in Design-Expert 13.1.

## 3. Results and Discussion

### 3.1. Extraction of MAAs from Two Macroalgae

Various factors have been reported to influence the extraction efficiency of MAAs. In this study, MAAs were extracted from *E. kurome* and *U. lactuca* using different liquid–solid ratios, temperatures, and separation times. As shown in Figure S2, the yield of MAA extract from *E. kurome* reached a maximum of 164.67 mg/g when the liquid–solid ratio, extraction temperature, and extraction time was 35:1 mL/g, 45 °C, and 120 min, respectively. As for *U. lactuca*, the highest yield of MAA extract was 151.11 mg/g when the liquid–solid ratio, extraction temperature and extraction time was 25:1 mL/g, 40 °C, and 120 min, respectively. The results of single factor experiments of MAAs from *U. lactuca* have been published previously in Chinese, so the data are no longer shown in Figure S2.

Response surface optimization has been widely used for identifying optimal process parameters due to its numerous advantages over single-factor experiments. To achieve the highest MAA extract yield, a Box–Behnken design experiment was adopted based on the aforementioned single-factor tests. The current Box–Behnken design included a total of 17 runs for optimizing three parameters: liquid–solid ratio, extraction temperature, and extraction time. The MAA extract yield was set as the response value. Multiple regression analysis was conducted on the experimental results shown in Table S6 using Design Expert 13.1.0.1 to obtain a polynomial equation described below, where Y represents the predicted MAA extract yield, and A, B, and C represent the liquid–solid ratio, extraction temperature, and extraction time, respectively. The significance and suitability of the model were analyzed using ANOVA, with the statistical information presented in Table S7. The model exhibited an F-value of 17.17 and a *p*-value of 0.0006, which was less than 0.01, indicating high significance. The F-value for the lack of fit was insignificant (*p* = 0.3009 > 0.05), confirming the model’s validity. The coefficient of determination (R^2^) was 0.9567. Additionally, the coefficient of variation (C.V.%) was 4.83, demonstrating the model’s high precision and excellent reliability.

Y = 170.64 + 7.33A − 7.07B + 6.35C + 14AB − 15.75AC − 1.15BC − 17.87A^2^ − 25.02B^2^ − 9.32C^2^ (6)

**Figure 1:**
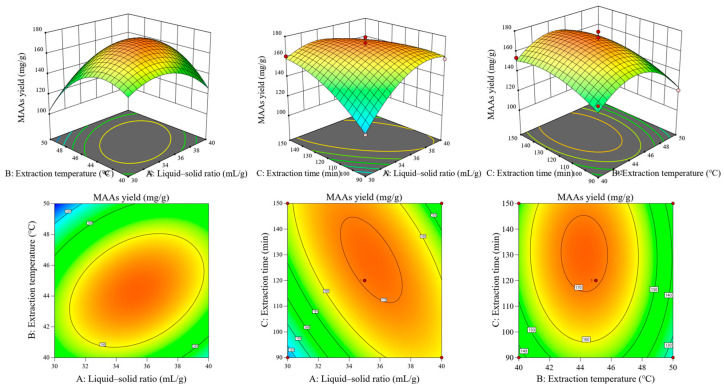
Response surface and contour plots showing the effect of different extraction parameters on the yield of MAA extract from *E. kurome*.

As shown in Table S7, the linear coefficients (A, B, C), the quadratic term coefficients (A2, B2, C2), and the cross product coefficients (AB, AC) were significant (*p* < 0.05), whereas the cross product coefficient (BC) had no significant effect on the MAA extract yield. Based on the F value of these factors, it can be concluded that the order of influence on the extraction of MAAs was A > B > C (liquid–solid ratio > extraction temperature > extraction time). Data were analyzed using Design-Expert 13.1.0.1 software, and the three-dimensional response surface and two-dimensional contour plots are shown in Figure 1. The predicted optimal extraction condition of MAAs was determined as follows: liquid–solid ratio, 34.925 mL/g; extraction temperature, 44.23 °C; and extraction time, 130.896 min. The predicted value of 172.282 mg/g was obtained from this model. Experiments were subsequently carried out to verify the predicted values. Practical extraction conditions were adjusted as follows: liquid–solid ratio, 35 mL/g; extraction temperature, 44 °C; and extraction time, 130 min. A practical value of MAA extract yield was obtained (169.71 ± 2.48 mg/g, n = 3), demonstrating the reliability of the model.

The RSM model was employed for the optimization of MAA extraction conditions from *U. lactuca*. Similarly to that of *E. kurome*, the liquid–solid ratio, extraction temperature, and extraction time were set as independent variables, while MAA extract yields were set as response values. The predicted optimal extraction condition from the RSM model was as follows: liquid–solid ratio, 25.768 mL/g; extraction temperature, 39.817 °C; and extraction time, 125.126 min, with a predicted value of 188.628 mg/g shown in Table S8. The adjusted extraction conditions (liquid–solid ratio, 26 mL/g; extraction temperature, 40 °C; extraction time, 125 min) were adopted in verification tests. The actual yield of MAA extract was 177.33 ± 5.27 mg/g, confirming the credibility of this model. These results have previously been published in Chinese, so they will not be repeated here. It can be concluded that the optimal extraction conditions vary depending on the species of algae. Among the above three factors, temperature had the greatest influence on MAA extraction efficiency from both *E. kurome* and *U. lactuca*. It was evident that the content of MAA extract in *U. lactuca* was greater than that in *E. kurome*, and both were higher than the content in *Bangia fusco-purpurea*, *Gelidium amansii*, and *Gracilaria confervoides* in our previous study [37].

### 3.2. Extraction of Glyceroglycolipids from Two Macroalgae

The effects of four factors (liquid–solid ratio, extraction temperature, methanol ratio, extraction time) on the extraction efficiency of glyceroglycolipids were investigated through single factor tests. It can be seen in Figure S3 that the glyceroglycolipid extract yield from *E. kurome* reached the highest (123.53 ± 2.03 mL/g) when the liquid–solid ratio, extraction temperature, methanol ratio, and extraction time were 25 mL/g, 45 °C, 70%, and 90 min, respectively. Similarly, the glyceroglycolipid extract yield from *E. kurome* was the highest (155.87 ± 3.89 mL/g) when the liquid–solid ratio, extraction temperature, methanol ratio, and extraction time were 15 mL/g, 55 °C, 70%, and 60 min, respectively.

**Figure 2:**
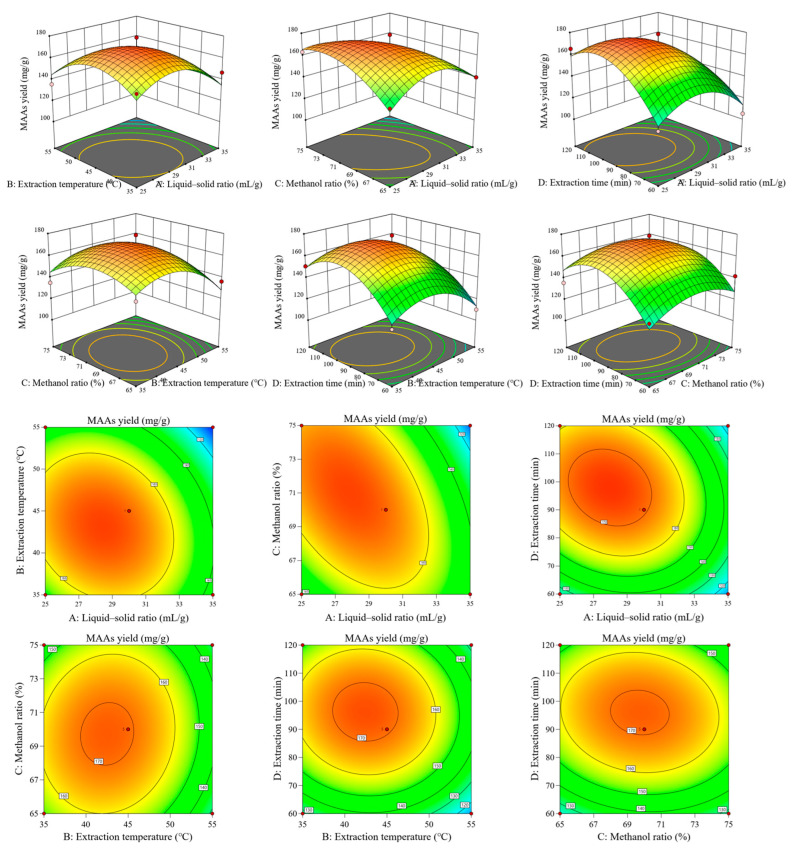
Response surface and contour plots showing the effect of different extraction parameters on the yield of glyceroglycolipid extract from *E. kurome*.

Based on the results from single-factor experiments, the Box–Behnken design was utilized to optimize the extraction conditions of glyceroglycolipids from *E. kurome*. Four factors (A: liquid–solid ratio; B: extraction temperature, C: methanol ratio, D: extraction time) were optimized, each with three levels. There were a total of 29 runs based on the Box–Behnken design with the glyceroglycolipid extract yield as the response variable, as shown in Table S9. The response surface and contour plots showing the effect of different extraction parameters on the yield of glyceroglycolipid extract from *E. kurome* are presented in Figure 2. As shown in Table S10, the model had F = 10.65 and was highly significant (*p* < 0.0001), while the lack of fit was not significant (*p* = 0.0652 > 0.05). Statistical information from ANOVA (Table S10) showed that the model had high credibility (R^2^ = 0.9142, R_Adj_^2^ = 0.8284, R_Pred_^2^ = 0.532, C.V.% = 6.3). The polynomial equation derived from this model was described as follows:

Y = 170.69 − 13.67A − 8.98B − 0.8275C + 9D − 6.82AB − 14.67AC − 7.9AD + 2.46BC − 1.91BD − 2.89CD − 19.51A^2^ − 18.35B^2^ − 13.29C^2^ − 22.94D^2^ (7)

Table S10 also suggests that the order of these four factors on extraction efficiency from *E. kurome* was A > D > B > C (liquid–solid ratio > extraction time > extraction temperature > methanol ratio). There was a significant impact of A, B, D, AC, A^2^, B^2^, C^2^, and D^2^, while other coefficients were not significant. The optimal conditions predicted by this model were as follows: liquid–solid ratio, 27.81 mL/g; extraction temperature, 43.343 °C; methanol ratio, 70.833%; and extraction time, 98.055 min, with a predicted response value of 175.566 mg/g. Experiments were carried out with the adjusted extraction conditions (liquid–solid ratio, 28 mL/g; extraction temperature, 43 °C; methanol ratio, 70%; extraction time, 98 min), obtaining an actual glyceroglycolipid extract yield of 163.51 ± 3.16 mg/g. These results suggested the excellent reliability of the model for optimization of glyceroglycolipid extract from *E. kurome*.

In order to obtain higher extraction efficiency of glyceroglycolipids from *U. lactuca*, the liquid–solid ratio (A), extraction temperature (B), methanol ratio (C), and extraction time (D) were optimized via response surface experiments. The response value obtained from 29 runs is presented in Table S11, and the ANOVA data are shown in Table S12. It can be seen that the model was extremely significant (*p* < 0.0001) and the lack of fit was not significant (*p* = 0.0955). Meanwhile, regression analysis data (R^2^ = 0.9689, R_Adj_^2^ = 0.9379, R_Pred_^2^ = 0.8328, C.V.% = 4.2) demonstrated the good credibility of this model. The multiple regression equation obtained from this model was as follows.

Y = 221.6 + 7.91A − 6.37B + 7.15C − 5.96D − 3.44AB + 5.49AC + 1.32AD + 0.8325BC − 7.96BD − 0.1375CD − 32.01A^2^ − 31.22B^2^ − 37.59C^2^ − 32.66D^2^ (8)

**Figure 3:**
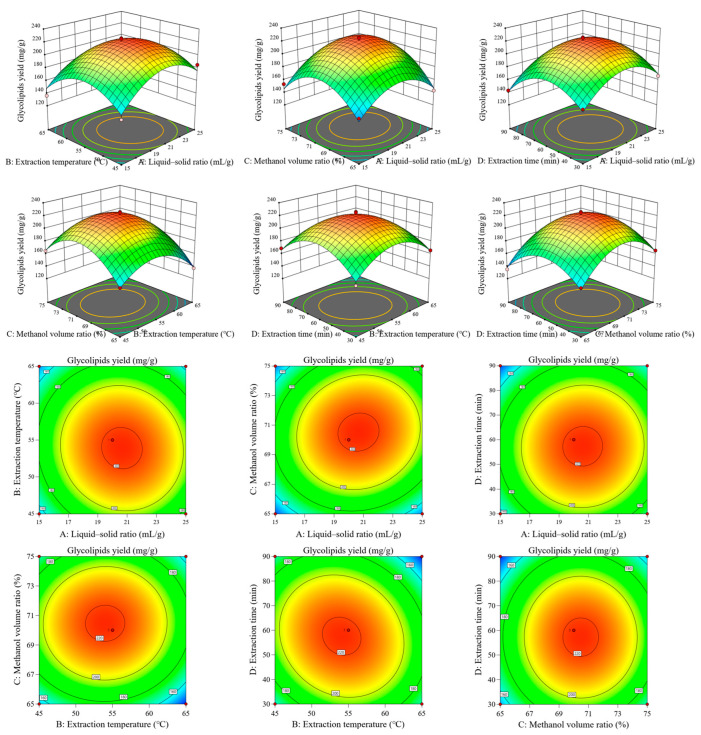
Response surface and contour plots showing the effect of different extraction parameters on the yield of glyceroglycolipid extract from *U. lactuca*.

Response surface and contour plots are presented in Figure 3. The order of the four factors on glyceroglycolipid extract yield was derived from the F value in Table S12. Similarly to MAA extraction from these two macroalgae, the liquid–solid ratio had the greatest impact on glyceroglycolipid extraction efficiency, followed by the methanol ratio, extraction temperature, and extraction time. There was a significant influence of the linear coefficients (A, B, C, D), the quadratic term coefficients (A^2^, B^2^, C^2^, D^2^), and cross product coefficients (BD) on glyceroglycolipid extract yield from *U. lactuca*. The best conditions predicted by this model (liquid–solid ratio, 20.682 mL/g; extraction temperature, 54.017 °C; methanol ratio, 70.523%; extraction time, 57.687 min) were adjusted to liquid–solid ratio, 20 mL/g; extraction temperature, 54 °C; methanol ratio, 70%; and extraction time, 58 min. The glyceroglycolipid extract yield measured by experiments was 213.45 ± 2.07 mg/g, approaching the predicted value of 223.06 mg/g, which demonstrated the reliability of this model for glyceroglycolipid extraction from *U. lactuca*. It can be concluded that the glyceroglycolipids from *U. lactuca* were more abundant than those from *E. kurome*, but both were lower than those from *B. fusco-purpurea* [38], indicating that these two macroalgae may not be the best sources of glyceroglycolipids. This study achieved the preparation of MAAs and glyceroglycolipids from marine macroalgae at the laboratory scale. However, scaling up the extraction process still faces several challenges, such as adapting laboratory conditions to industrial equipment, ensuring a consistent supply of high-quality raw materials, and maintaining cost-effectiveness. Additionally, the process will need further optimization for regulatory compliance and environmental sustainability.

### 3.3. Antioxidant Activity and Applications of MAA and Glyceroglycolipid Extracts

#### 3.3.1. Determination of Antioxidant Properties of Different Extracts

**Figure 4:**
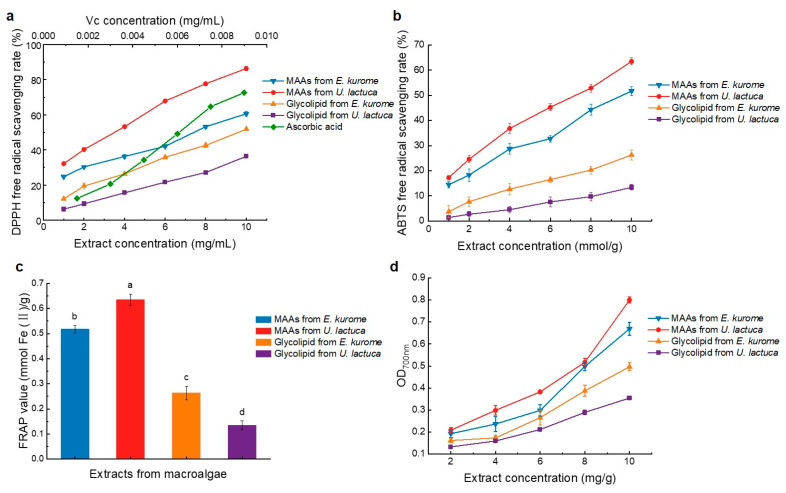
Antioxidant capacity ((**a**): DPPH free radical scavenging ability; (**b**): ABTS free radical scavenging ability; (**c**): iron reduction ability; (**d**): potassium ferricyanide reducing capacity) of MAA and glyceroglycolipid extract from two macroalgae.

It is well known that MAAs are strong contenders for an eco-friendly UV protector. In addition, MAAs have been reported to possess a variety of biological activities, such as antioxidant, anti-inflammatory, wound-healing, anti-photoaging, cell-proliferation-stimulating and anti-cancer properties [39]. Therefore, the antioxidant activities of MAA and glyceroglycolipid extracts obtained from macroalgae were evaluated by DPPH [40], ABTS, FRAP and potassium ferricyanide assay. As shown in Figure 4a, MAA and glyceroglycolipid extracts from *E. kurome* and *U. lactuca* showed different scavenging abilities for DPPH, with strong dose-dependency. With the gradual increase in extract concentration, the percentage of DPPH free radical scavenging also increased. In the concentration range of 1–10 mg/mL, the scavenging rates of MAA extract from *E. kurome*, MAA extract from *U. lactuca*, glyceroglycolipid extract from *E. kurome*, and glyceroglycolipid extract from *U. lactuca* ranged from 24.67% to 60.61%, 32.16% to 86.33%, 12.11% to 51.86%, and 6.23% to 36.33%, respectively. MAAs from *U. lactuca* had a stronger scavenging capacity for DPPH free radicals than that from *E. kurome*, while the scavenging ability of glyceroglycolipid extract from *E. kurome* was stronger than that from *U. lactuca*, but all values were lower than those of ascorbic acid. The IC_50_ value of the extract clearance rate was then obtained, representing the extract concentration required to achieve a scavenging rate of 50%. According to Table 1, the DPPH scavenging ability of MAA extract from *U. lactuca* was significantly stronger than other extracts, with an IC_50_ value of 3.59 mg/mL, followed by MAA extract from *E. kurome* (7.43 mg/mL). The scavenging abilities of glyceroglycolipid extract from *E. kurome* and *U. lactuca* were relatively moderate, with IC_50_ values of 9.89 mg/mL and 14.62 mg/mL, respectively.

It can be seen from Figure 4b and Table 1 that the scavenging ability of different extracts on ABTS free radicals was consistent with that of DPPH free radicals. MAA extract from *U. lactuca* had the strongest scavenging ability on ABTS free radicals (IC_50_ = 7.17 mmol/g). Their active functional groups can be fully dissolved to contact and react with free radicals. MAAs like porphyra-334 and shinorine effectively inhibited free radicals through hydrogen atom transfer, supporting their significant in vitro antioxidant potential. Based on this characteristic, it can be inferred that MAA extract from *U. lactuca* can not only remove active oxygen efficiently but also combine with other molecules inside the algae cells, generating sub-derivatives which can activate various antioxidant enzymes such as superoxide dismutase (SOD) and catalase (CAT). Likewise, enhanced transcriptional regulation was only promoted by MAAs in cells after exposure to UVR-induced oxidative stress [41]. In this study, the FRAP assay was used to assess the antioxidant abilities of different extracts. As shown in Figure 4c, the iron ion reduction ability varied significantly among the different extracts. The MAA extract from *U. lactuca* exhibited the highest iron ion reduction capacity (2.651 mmol Fe^2+^/g), suggesting that MAA extracts not only scavenge free radicals but also donate hydrogen atoms, thereby demonstrating good antioxidant performance [42]. Figure 4d showed that the total reducing capacity of MAA and glyceroglycolipid extracts increased with concentration, demonstrating a strong concentration dependence in the range of 1–10 mg/mL. The MAA extract from *U. lactuca* had the highest total reducing capacity, with an absorbance value of 0.80 at 700 nm. In conclusion, the MAA extract from *U. lactuca* demonstrated the best performance in various antioxidant evaluations, indicating significant potential as a food antioxidant.

As compounds whose structures are known, porphyra-334 and shinorine have been reported to have good antioxidant properties. Although their antioxidant activity in vitro was lower than that of ascorbic acid, their antioxidant ability to inhibit free radicals through hydrogen atom transfer was considerable [41]. In recent years, an increasing number of MAAs and their derivatives with different structures have been confirmed to have significant antioxidant activities [43], such as MAA precursors (gadusol with higher than 30% inhibition rate of ABTS+ at 8 μM) [44,45], mono-substituted MAAs [8,46], and di-substituted MAAs [47]. Regarding the DPPH radical scavenging activity, the IC_50_ value of mycosporine-glycine was 43 µM, while that of mycosporine-2-glycine was 22 µM. In contrast, the IC_50_ values of porphyra-334 and shinorine were 185.2 and 399 µM, respectively. As for the ABTS radical scavenging activity, the IC_50_ value of mycosporine-glycine, porphyra-334 and shinorine was 3, 133, and 94 µM, respectively. These IC_50_ values were lower than those of extracts in this study. This difference was attributed to the use of crude extracts for antioxidant activity testing, which likely resulted in higher IC_50_ values.

#### 3.3.2. Antioxidant Properties of Flaxseed Oil Protected by MAA Extract

**Figure 5:**
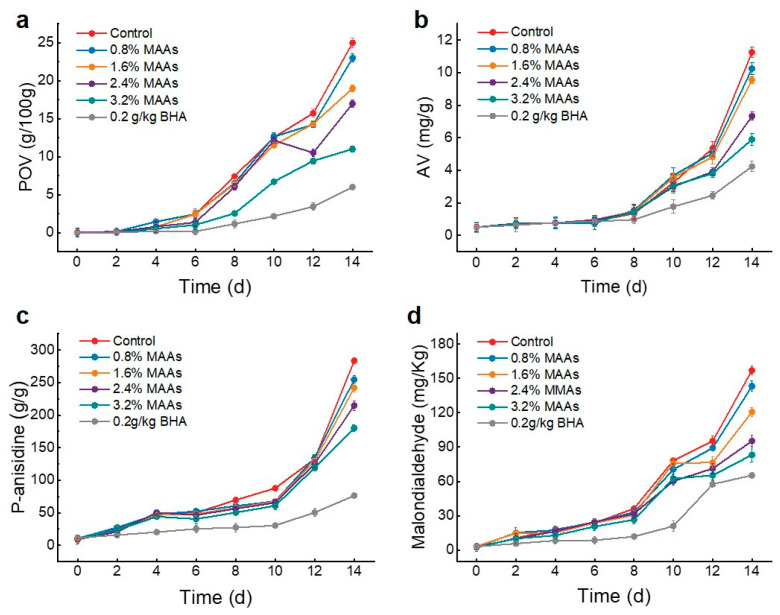
Effects of MAA extracts on POV (**a**), AV (**b**), P-anisidine (**c**) and malondialdehyde (**d**) of flaxseed oil.

POV is widely recognized as an early indicator of oil rancidity and a crucial metric for assessing oil quality [48]. A significant increase in POV indicates reduced oil stability and the onset of oxidative rancidity. Given their superior antioxidant properties, the MAA extract from *U. lactuca* was selected to assess its efficacy in protecting flaxseed oil from oxidation. As shown in Figure 5a, the POV of samples in different groups gradually increased with extended storage time. The MAA extract from *U. lactuca* demonstrated a significant dose-dependent effect in inhibiting flaxseed oil oxidation. Increasing the concentration of MAA extract corresponded to a reduction in the oxidation level of flaxseed oil, demonstrating the robust antioxidant efficacy of the MAA extract from *U. lactuca*. Oil deterioration is a multifaceted process encompassing numerous chemical reactions and physical transformations, and POV is just one of several indices used to gauge oil oxidation. As oil oxidation progresses, hydroperoxide decomposition or polymerization can occur, leading to rancidity. To comprehensively assess oil rancidity due to oxidation, AV [49] was introduced to quantify the free fatty acid content accurately. As shown in Figure 5b, the AV of flaxseed oil in the control group continuously increased with storage time, whereas the AV in the experimental group increased more slowly due to the addition of MAA extract. After 14 d of storage, the AV of samples containing 3.2% MAA extract (5.89 mg/g) decreased by 47.6% compared to the control (11.25 mg/g), highlighting the significant role of MAA extract in mitigating oil rancidity. Nonetheless, MAA extract exhibited lower efficacy in inhibiting oil rancidity compared to BHA, which yielded an AV of 4.23 mg/g.

P-anisidine, as a symbolic end-product of oil oxidation, is commonly used to determine oil oxidation and rancidity. In this study, the P-anisidine content in flaxseed oil treated with BHA and various concentrations of MAA extract was measured during storage. As shown in Figure 5c, when storage time exceeded 10 d, the P-anisidine content in flaxseed oil with different concentrations of MAA extract began to rise rapidly, indicating continuous intensification of oxidative rancidity. Notably, on the 14 d of storage, the P-anisidine content in the control reached 283.56 ± 6.48 g/g, significantly higher than in the treatment group with 3.2% MAA extract (179.99 ± 5.24 g/g) and the positive control group (76.34 ± 2.98 g/g). These results further emphasized the significant effect of MAA extract in delaying oxidative rancidity in flaxseed oil. Additionally, the action of free radicals on lipids leads to peroxidation, producing MDA as an end product. To thoroughly investigate the degree of oxidative rancidity in oils, MDA content was measured. As shown in Figure 5d, the MDA content in flaxseed oil with different MAA extracts exhibited a steady and slow rise within 8 d, followed by severe oxidative deterioration and a rapid increase in MDA content after 8 d. Fortunately, the rate of increase in MDA content in the MAA extract treatment group was lower than in the control group. After 14 d of storage, the MDA content in flaxseed oil supplemented with 3.2% MAA extract (83.19 ± 6.22 mg/kg) and BHA (65.32 ± 1.67 mg/kg) was significantly lower than in the control group (157.11 ± 4.18 mg/kg). In summary, the MAA extract from *U. lactuca* demonstrated significant activity in protecting oil from oxidation in a concentration-dependent manner. Similarly, Coba et al. [45] explored the antioxidative activity and inhibition of lipid peroxidation in a water-soluble medium by MAAs in vitro, finding that the hydrosoluble radical scavenging activity of MAA extract was dose-dependent. Compared to glyceroglycolipids, MAAs was also more susceptible to oxidation, making it a superior antioxidant because it has a lower redox potential and can donate an electron to stabilize free radicals [39]. Furthermore, MAA extract also plays an important antioxidant role in living organisms. Yakovleva et al. [50] concluded that the biological antioxidant mycosporine-glycine played a crucial role in coral tissue and zooxanthellae in ensuring coral reefs survive under thermal stress. These studies suggest that MAAs and their derivates from these two macroalgae have potential in the fields of food preservation and antioxidation in the near future. For example, MAAs could be blended with curing salts or injected into meat emulsions during the production process, reducing the need for synthetic antioxidants like BHA or butylated hydroxytoluene (BHT). This application would not only improve the oxidative stability of the meat but also meet consumer preferences for natural additives. In fruit and vegetable preserves, such as jams, jellies, or pickled products, MAAs could help maintain the natural color, flavor, and nutritional value by preventing oxidative deterioration of pigments like anthocyanins or carotenoids, which could provide an eco-friendly alternative to synthetic antioxidants.

### 3.4. Moisturizing Activity and Applications of MAA and Glyceroglycolipid Extracts

#### 3.4.1. Moisturizing Absorption and Moisturizing Retention Ability of Different Extracts

**Figure 6:**
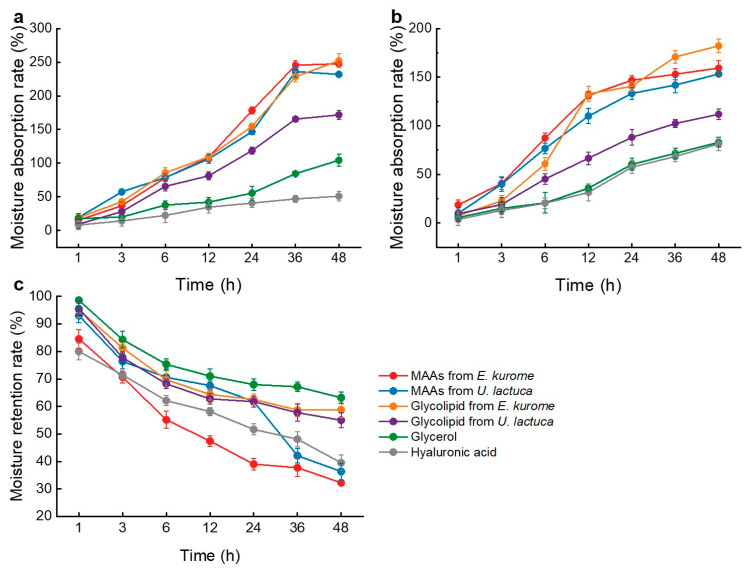
Moisture absorption rate ((**a**): RH = 43%; (**b**): RH = 81%) and moisture retention rate in dry conditions (**c**) of the extracts from *E. kurome* and *U. lactuca*.

In addition to their antioxidant activity, various compounds from marine macroalgae possess excellent properties such as anti-ultraviolet, moisturizing, and anti-inflammatory activities. In recent years, there have been increasing reports on active substances derived from macroalgae as moisturizers. Therefore, this study aimed to evaluate the hygroscopic and moisturizing activities of the different extracts mentioned above. The hygroscopic activities of MAA extract from *E. kurome*, MAA extract from *U. lactuca*, glyceroglycolipid extract from *E. kurome*, glyceroglycolipid extract from *U. lactuca*, glycerol, and HA were estimated under three different environmental humidity conditions of RH 43% and RH 81%. Then, the hydrating activities of these compounds were measured in a dry environment. As shown in Figure 6a, both MAA and glyceroglycolipid extracts demonstrated good hygroscopic properties at RH 43%, superior to glycerol and HA. The maximum moisture absorption rates were glyceroglycolipid extract from *E. kurome* > MAA extract from *E. kurome* > MAA extract from *U. lactuca* > glyceroglycolipid extract from *U. lactuca* > glycerol > HA. When the ambient humidity increased to RH 81%, the hygroscopic ability of MAA extract from *E. kurome* was the most significant in the first 12 h (Figure 6b). Over time, the hygroscopicity of glyceroglycolipid extract from *E. kurome* increased continuously, surpassing that of MAA extract. The maximum moisture rate reached 182.28 ± 7.189%, while that of glycerol was only 81.48 ± 7.169%. Compared to MAAs, glyceroglycolipids can not only combine with oxygen in water molecules to form hydrogen bonds through numerous hydrophilic groups but also form a network structure through interactions between sugar groups, effectively trapping water molecules and exhibiting excellent moisturizing activity. Under dry conditions, glycerol had the highest moisture retention rate, followed by glyceroglycolipid extract and HA. In contrast, the moisturizing activity of MAA extract was relatively weak, especially after 24 h (Figure 6c). After 48 h of dry treatment, the moisture rate of MAA extract from *U. lactuca* decreased to 36.36 and 32.26%. Fortunately, the hydration rate of glyceroglycolipid extract remained relatively high after 48 h, at 58.79 and 55.00%, respectively. Our previous study discovered that MAA extracts from four species of red macroalgae exhibited good hygroscopic ability. Various fatty acids from *Cladophora glomerata*, a filamentous green microalga found in marine environments, may act as an emollient and protect skin from excessive water loss [51]. Additionally, it was possible to isolate a sulfated polysaccharide from this alga, suggesting a potential moisturizing effect [52]. Glyceroglycolipids, as complex lipids composed of hydrophilic sugar groups and hydrophobic fatty acid chains, could enhance skin barrier function and reduce transepidermal water loss. These dual characteristics contribute to their moisturizing effects, which are beneficial for skincare and pharmaceutical applications. These studies indicate that extracts from *E. kurome* and *U. lactuca* have promising prospects as moisturizing agents.

#### 3.4.2. Preparation and Characterization of Water-Retaining Agent from Glyceroglycolipid Extract

In recent years, seaweed extracts have increasingly been used in the preparation of biological water-retaining agents. For example, a new biological water-retaining agent developed using poly-glutamate and seaweed extract demonstrated the ability to maintain water, resist salt, and promote crop growth, showing significant application potential and development prospects [53]. The characteristics of various extracts mentioned above indicated that the glyceroglycolipid extract from *E. kurome* exhibited excellent moisture retention. Therefore, it was selected to prepare a biological water-retaining agent in this study. The amounts of acrylic acid, initiator content, crosslinking agent dosage, and neutralization degree of acrylic acid were optimized through single-factor experiments. As shown in Figure S4a, when the mass ratio of acrylic acid to glyceroglycolipid extract was adjusted to 2:1, the water absorption performance was optimal, with a water absorption ratio of 665.57 ± 11.54 g/g. When the initiator content was 1.4%, the water absorption ratio reached a peak of 604.85 ± 18.83 g/g (Figure S4b). With the increase in crosslinking agent content, the hygroscopic ratio initially increased and then decreased, reaching its highest value (714.43 ± 3.68 g/g) at 0.20% crosslinking agent content (Figure S4c). When the neutralization degree of acrylic acid was 65%, the water absorption ratio increased to 755.92 ± 4.92 g/g (Figure S4d). From these single-factor tests, the optimal levels of several factors were determined. Subsequently, the optimal conditions for preparing the biological water-retaining agent were established through an orthogonal test as outlined in Table S13. It can be concluded that the order of the four factors affecting the water-retaining agent was B > A > C > D (initiator > mass ratio of acrylic acid > crosslinking agent > neutralization degree of acrylic acid). The moisture absorption ratio of the prepared water-retaining agent was 825.47 g/g under the optimal conditions (a mass ratio of acrylic acid to glyceroglycolipid extract of 1:1, initiator content of 1.4%, crosslinking agent content of 0.2%, and neutralization degree of acrylic acid of 60%).

**Figure 7:**
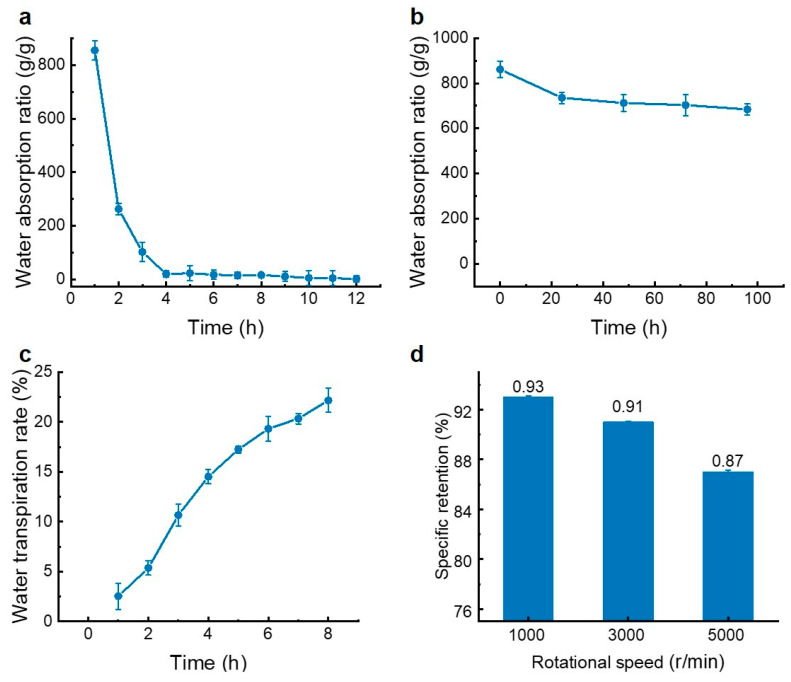
Different properties of prepared water-retaining agent. (**a**): Water absorption rate at different times; (**b**): effect of high temperature on water absorption rate at different times; (**c**): anti-pressure ability of the prepared water-retaining agent; (**d**): effect of refrigeration on water absorption rate at different times.

Then, the properties of the prepared water-retaining agent were tested. As shown in Figure 7a, during the water absorption process, the peak water absorption rate mainly occurred within the first hour. Over time, the water absorption rate gradually slowed until it approached zero at 4 h. At 60 °C, the cumulative water transpiration rate of the water-retaining agent increased slowly with time (Figure 7b), indicating excellent high-temperature resistance. With increased freezing time at −20 °C, the water absorption ratio of the water-retaining agent showed a slight downward trend (Figure 7c), but it still remained over 500 g/g, suggesting good freezing resistance. As shown in Figure 7d, the water retention decreased with increasing pressure, but the change was not significant, indicating good pressure resistance. Recently, various polysaccharides from plants and marine organisms have exhibited great moisture-retention abilities due to their sulfated groups, which are main active sites for moisture absorption and retention. Chitin, chitosan, and their derivatives, as classic moisturizers, have been thoroughly studied for their moisture absorption and retention abilities [54,55]. Jesumani et al. found that polysaccharides extracted from seaweed showed excellent moisture absorption (52.1%) and retention (63.24%) abilities [56]. In vitro moisturizing assays showed that the moisturizing rate of polysaccharides from *E. prolifera* reached about 98%, which was closely related to that of HA. [57]. The study showed that incorporating seaweed extracts into natural polymers showed applicational potential in future food packaging. For example, a κ-CG/chitosan-based film containing allyl isothiocyanate showed that the oppositely charged polysaccharide film had good coating properties [58]. These studies revealed that the sources of natural biological moisturizers are extensive and need further development and utilization. Currently, most water-retaining agents, both domestically and internationally, are prepared from polysaccharides such as hyaluronic acid. Few studies have reported the feasibility of glyceroglycolipids as water-retaining materials. In this study, glyceroglycolipid extracts from *E. kurome* and *U. lactuca* were used as a raw material to prepare a water-retaining agent and its properties were evaluated, promoting the further utilization of marine resources.

### 3.5. Purification and Identification of MAAs and Glyceroglycolipids from Macroalgae

According to preliminary yield and target activity screening, the yield and activity of MAA and glyceroglycolipid extract from *U. lactuca* were superior to those from *E. kurome*. Therefore, a silica gel column chromatography method for the separation of MAA and glyceroglycolipid extracts was established. The structure was identified using thin layer chromatography (TLC) [59], ultraviolet/infrared spectroscopy, and HPLC [60]. UV spectrum scanning showed that the crude MAA extract from *U. lactuca* had significant absorption in the range of 310–330 nm, which was the characteristic absorption range of MAAs. Subsequently, MAAs were purified and identified from the crude extract by HPLC and ESI-MS [61]. The MAA extract from *U. lactuca* exhibited significant absorption at 330 nm, with prominent retention times at 3.037 min and 3.56 min. Based on HPLC and ESI-MS results, fraction H2 isolated from silica gel column chromatography was identified as shinorine and porphyra-334 [62,63]. Quantitative analysis revealed that the average MAA content in the crude extract was 58.33 mg/g, indicating that the MAA content in *U. lactuca* could account for 10.32 mg/g of the dry powder. The detailed data have already been published in Chinese and will not be shown here.

**Figure 8:**
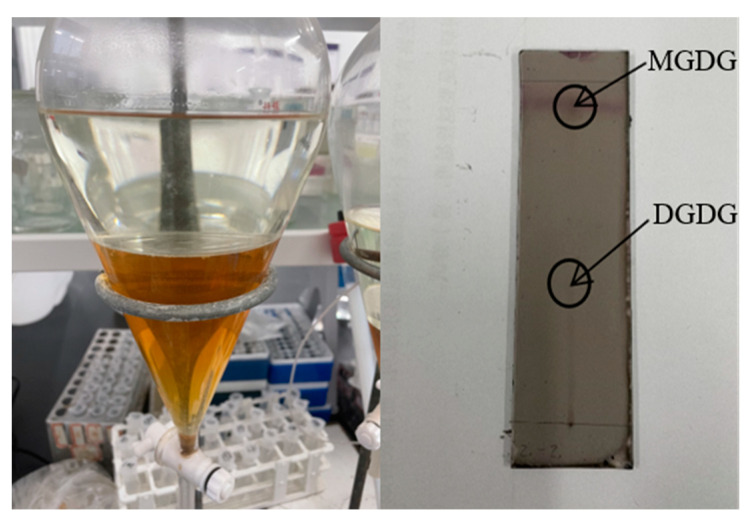
Liquid–liquid extraction and TLC detection of glyceroglycolipids from *U. lactuca*.

The glyceroglycolipids in the methanol extract were enriched using a chloroform–methanol mixed system. There was 5.1 g of total fat obtained from the glyceroglycolipid solution after extraction, accounting for about 17% of the dried MAA crude extract. Subsequently, 10.32 mg of purified glyceroglycolipids was obtained through silica gel column chromatography, a method widely used in the separation of various glyceroglycolipids from *Chondria armata* [13], *Laminaria japonica* [22], and *U. lactuca* [27]. As shown in Figure 8, in the presence of chromogenic agents, glyceroglycolipid compounds exhibited obvious red and purple spots on the silica gel plate, which could serve as an important basis for the initial identification of glyceroglycolipids. The Rf values of the two spots were 0.93 and 0.49, respectively, suggesting that the products were non-polar esters and MGDG or DGDG, according to our previous study [38].

**Figure 9:**
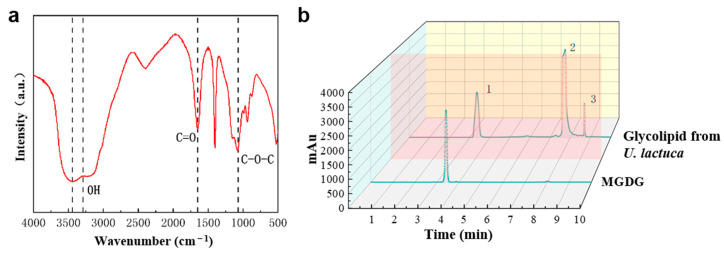
Infrared spectrogram (**a**) and HPLC detection (**b**) of glyceroglycolipid extracts from *U. lactuca*.

Infrared spectroscopic analysis was employed to further determine the chemical structure of the purified products. From Figure 9a, the infrared spectrum can be divided into two parts: the 4000–1350 cm^−1^ region, which was the absorption band generated by stretching vibrations, and the 1350–650 cm^−1^ region, which was the fingerprint region. The broad and high peaks at 3447.79 and 3293.78 cm^−1^ were attributed to O-H stretching and vibration. The peak near 1700 cm^−1^ indicated the absorption of C=O in the low wave region due to conjugation with C=C, consistent with the characteristic peak of esters. The peak at 1043 cm^−1^ corresponded to the asymmetrical expansion of the C-O-O ether bond on the sugar ring, and the absorption peak at 929.32 cm^−1^ indicated the β-glucoside bond, which aligned with the characteristic peak of the sugar group. As shown in Figure 9b, the purified glyceroglycolipids had characteristic absorption at 210 nm. Additionally, the absorption peak at 3.233 min matched that of the MGDG standard substance (3.420 min) within the acceptable error range (±0.2 min), suggesting it could be identified as MGDG.

The extraction methodology developed in this study for MAAs and glyceroglycolipids from *E. kurome* and *U. lactuca* can be adapted to other macroalgae species, with potential adjustments in extraction conditions to accommodate species-specific biochemical profiles. These extracted compounds hold promise for incorporation into functional foods, edible packaging, and cosmetics. For example, MAAs could enhance the oxidative stability of oils and glyceroglycolipids could improve the moisture content of various food products. Additionally, these compounds could be used in edible packaging to preserve food while providing antioxidant benefits. While the initial results are promising, further research is needed to optimize the stability, bioavailability, and cost-effectiveness of these compounds for large-scale industrial applications, as well as to address regulatory and consumer acceptance challenges.

## 4. Conclusions

This study developed an efficient method for extracting MAAs and glyceroglycolipids from marine macroalgae *E. kurome* and *U. lactuca*, demonstrating their promising biological activities. The results revealed that MAAs exhibit significant antioxidant potential, with applications in reducing food oxidation, thereby potentially increasing shelf life. For example, the antioxidant activity of MAAs was successfully applied to protect flaxseed oil from oxidative damage. Natural antioxidants like MAAs hold application potential in food and cosmetic fields, but their industrial adoption is hindered by several challenges. These include issues with stability, as the compounds are sensitive to factors like heat and light, leading to reduced effectiveness over time. Additionally, the high costs of extraction and purification processes, as well as the scalability of these methods, pose significant barriers for large-scale production. The MAA and glyceroglycolipid extracts showed strong antioxidant and moisturizing properties, making them suitable for use in cosmetics and as humectants in skincare products. In preserves and other processed foods, glyceroglycolipids, with their ability to retain moisture, could be used to improve the texture and mouthfeel, especially in low-fat or reduced-calorie products where moisture retention is critical. These compounds can also be used in salad dressings or sauces, where they can act as natural emulsifiers to improve stability and texture while providing health benefits. Further research is needed to ensure the stability, safety, and efficacy of these compounds in food formulations, but their potential for enhancing both product quality and shelf life is considerable.

However, several limitations need to be addressed for the broader application of these compounds. The scalability of the extraction process remains a major challenge, as large-scale production may face issues such as lower yields, higher solvent usage, and increased operational costs. Moreover, the stability of these compounds, particularly their antioxidant efficacy over time and under varying environmental conditions, must be improved for long-term use in industrial products. Future studies should focus on validating the optimized condition, developing stabilization strategies, including encapsulation techniques, and assessing the economic viability of scaling up the extraction process. Despite these challenges, this research contributes to the growing field of marine resource utilization, offering new possibilities for the development of eco-friendly products in the food, cosmetics, and pharmaceutical industries. Further investigations into industrial-scale production, long-term stability, and the real-world performance of these compounds are essential for realizing their full potential and addressing the increasing demand for sustainable, natural alternatives under industrial conditions.

## Figures and Tables

**Table 1:** IC_50_ value of DPPH and ABTS+ free radical capacity of different extracts.

Free Radicals	Extracts	Regression Equation	IC_50_
DPPH	MAA extract from *E. kurome*	y = 0.0390x + 0.2104, R^2^ = 0.9903	7.43 mg/mL
	MAA extract from *U. lactuca*	y = 0.0609x + 0.2811, R^2^ = 0.9900	3.59 mg/mL
	Glyceroglycolipid extract from *E. kurome*	y = 0.0404x + 0.1006, R^2^ = 0.9903	9.89 mg/mL
	Glyceroglycolipid extract from *U. lactuca*	y = 0.0324x + 0.0264, R^2^ = 0.9937	14.62 mg/mL
ABTS+	MAA extract from *E. kurome*	y = 0.0412x + 0.1041, R^2^ = 0.9907	9.61 mmol/g
	MAA extract from *U. lactuca*	y = 0.0236x + 0.0232, R^2^ = 0.9906	20.20 mmol/g
	Glyceroglycolipid extract from *E. kurome*	y = 0.0496x + 0.1445, R^2^ = 0.9909	7.17 mmol/g
	Glyceroglycolipid extract from *U. lactuca*	y = 0.0130x − 0.0016, R^2^ = 0.9900	38.58 mmol/g

## Data Availability

The original contributions presented in this study are included in the article/Supplementary Material. Further inquiries can be directed to the corresponding author.

## Supplementary Materials

The following supporting information can be downloaded at: https://www.mdpi.com/article/10.3390/foods14030440/s1, Figure S1: Extraction process optimization of MAAs and glyceroglycolipids from macroalgae; Figure S2: Effects of liquid–solid ratio (a), extraction temperature (b), and extraction time (c) on the yield of MAA extract from *E. kurome*; Figure S3: Effects of liquid–solid ratio (a), extraction temperature (b), methanol volume ratio (c), and extraction time (d) on the yield of glyceroglycolipid extract from *E. kurome* and *U. lactuca*; Figure S4: Effects of mass ratio of acrylic acid to glyceroglycolipid extract (a), initiator content (b), crosslinking agent content (c), and neutralization of acrylic acid (d) on the water absorption ratio of prepared biological water-retaining agents; Table S1: Factors and levels in single factor tests of MAA extraction from *E. kurome* and *U. lactuca*; Table S2: Box–Behnken design factors and horizontal coding of MAA extraction process from *E. kurome* and *U. lactuca*; Table S3: Factors and levels in single factor tests of glyceroglycolipid extraction from *E. kurome* and *U. lactuca*; Table S4: Box–Behnken design factors and horizontal coding of glyceroglycolipid extraction process from *E. kurome* and *U. lactuca*; Table S5: Factors and levels for orthogonal test of water retainer preparation; Table S6: Response surface test design and results of MAA extraction from *E. kurome*; Table S7: Significance tests and analysis of variance for the regression model of MAA extract from *E. kurome*; Table S8: Response surface test design and results of MAA extraction from *U. lactuca*; Table S9: Response surface test design and results of glycolipids extraction from *E. kurome*; Table S10: Significance tests and analysis of variance for the regression model of glyceroglycolipid extract from *E. kurome*; Table S11: Response surface test design and results of glycolipids extraction from *U. lactuca.* Table S12: Significance tests and analysis of variance for the regression model of glyceroglycolipid extract from *U. lactuca;* Table S13. Results and analysis of orthogonal experiment for process optimization of water-retention agent preparation.
