# Supplementary material for: Comprehensive Extraction and Biological Activities of Mycosporine-like Amino Acids and Glyceroglycolipids Extracts from Two Macroalgae Ecklonia kurome and Ulva lactuca
Source: Foods. 2025 Jan 29;14(3):440. doi: 10.3390/foods14030440 (PMC11817109; doi:10.3390/foods14030440)
Supplement: Supplementary file 1 [file foods-14-00440-s001.zip › Supplementary tables.pdf]

Table S1 Factors and levels in single factor tests of MAAs extraction from *E. kurome* and *U. lactuca*

Factors	Levels					Fixed factors
Liquid-solid ratio (mL/g)	20:1	25:1	30:1	35:1	40:1	45 ℃ of extraction temperature and 120 min of extraction time
Extraction temperature (℃)	35	40	45	50	55	25:1 mL/g of liquid-solid ratio and 120 min of extraction time
Extraction time (min)	60	90	120	150	180	25:1 mL/g of liquid-solid ratio and 40 ℃ of extraction temperature

Table S2 Box-Behnken design factors and horizontal coding of MAAs extraction process from *E. kurome* and *U. lactuca*

Macroalgae	Factors	Levels		
		-1	0	1
E. kurome	A-Liquid-solid ratio (mL/g)	30	35	40
	B-Extraction temperature (℃)	40	45	50
	C-Extraction time (min)	90	120	150
U. lactuca	A-Liquid-solid ratio (mL/g)	20	25	30
	B-Extraction temperature (℃)	35	40	45
	C-Extraction time (min)	90	120	150

Table S3 Factors and levels in single factor tests of glyceroglycolipids extraction from *E. kurome* and *U. lactuca*

Factors	Levels					Fixed factors
Liquid-solid ratio (mL/g)	10:1	15:1	20:1	25:1	30:1	55 ℃ of extraction temperature, 65% of methanol volume ratio and 60 min of extraction time
Extraction temperature (℃)	35	45	55	65	75	30:1 mL/g of liquid-solid ratio, 65% of methanol volume ratio and 60 min of extraction time

Methanol volume ratio (%)	55	60	65	70	75	30:1 mL/g of liquid-solid ratio, 45 ℃ of extraction temperature and 60 min of extraction time
Extraction time (min)	30	60	90	120	150	30:1 mL/g of liquid-solid ratio, 45 ℃ of extraction temperature and 70% of methanol volume ratio

Table S4 Box-Behnken design factors and horizontal coding of glyceroglycolipids extraction process from *E. kurome* and *U. lactuca*

Macroalgae	Factors	Levels		
		-1	0	1
E. kurome	A-Liquid-solid ratio (mL/g)	25	30	35
	B-Extraction temperature (℃)	35	45	55
	C-Methanol ratio (%)	65	70	75
	C-Extraction time (min)	60	90	120
U. lactuca	A-Liquid-solid ratio (mL/g)	15	20	25
	B-Extraction temperature (℃)	45	55	65
	C-Methanol ratio (%)	65	70	75
	C-Extraction time (min)	30	60	90

Table S5 Factors and levels for orthogonal test of water retainer preparation.

Levels	Factors			
	acrylic acid/ glycolipid (g/g)	initiator content (%)	cross-linking agent content (%)	neutralization degree of acrylic acid (%)
1	1:1	1.1	0.15	55
2	1:2	1.4	0.2	60
3	1:3	1.7	0.25	65

Table S6 Response surface test design and results of MAAs extraction from *E. kurome*

Run	A (Liquid-solid ratio)	B (Extraction temperature)	C (Extraction time)	MAAs yield (mg/g)
1	-1	0	1	160.69±4.82
2	0	-1	-1	142.11±3.83
3	0	0	0	166.83±4.17
4	0	1	-1	121.00±2.79
5	0	1	1	128.24±3.04
6	0	0	0	173.61±2.67
7	-1	-1	0	136.19±2.55
8	0	-1	1	153.92±3.18
9	1	0	1	142.23±3.23
10	1	1	0	147.40±3.71
11	-1	0	-1	113.21±3.42
12	-1	1	0	103.23±3.21
13	1	0	-1	157.82±2.73
14	0	0	0	179.24±4.31
15	0	0	0	170.20±3.91
16	0	0	0	163.35±4.17
17	1	-1	0	124.37±3.20

Table S7 Significance tests and analysis of variance for the regression model of MAAs extract from *E. kurome*.

Source	Sum of Squares	df	Mean Square	F-value	p-value
Model	7703.99	9	856	17.17	0.0006**
A-Liquid-solid ratio	429.25	1	429.25	8.61	0.0219*
B-Extraction temperature	400.44	1	400.44	8.03	0.0253*
C-Extraction time	322.58	1	322.58	6.47	0.0385*
AB	784	1	784	15.73	0.0054**
AC	992.25	1	992.25	19.9	0.0029**

BC	5.29	1	5.29	0.1061	0.7541
A^2	1344.58	1	1344.58	26.97	0.0013**
B^2	2635.79	1	2635.79	52.87	0.0002**
C^2	365.74	1	365.74	7.34	0.0303*
Residual	348.98	7	49.85		
Lack of Fit	196.41	3	65.47	1.72	0.3009
Pure Error	152.57	4	38.14		
Cor Total	8052.98	16			

Table S8 Response surface test design and results of MAAs extraction from *U. lactuca*

Run	A (Liquid-solid ratio)	B (Extraction temperature)	C (Extraction time)	MAAs yield (mg/g)
1	0	-1	1	157.27±3.57
2	0	0	0	189.33±3.79
3	-1	0	1	147.65±4.32
4	0	-1	-1	147.60±3.57
5	0	1	1	154.25±2.89
6	0	0	0	187.71±3.04
7	0	0	0	193.47±4.36
8	1	1	0	146.46±5.01
9	1	0	-1	150.99±3.55
10	0	0	0	185.23±3.62
11	-1	1	0	148.71±4.51
12	1	-1	0	156.16±4.75
13	1	0	1	168.35±2.73
14	0	0	0	183.25±4.21
15	0	1	-1	146.27±5.33
16	-1	-1	0	140.50±3.66
17	-1	0	-1	143.50±4.21

Table S9 Response surface test design and results of glycolipids extraction from *E. kurome*

Run	A (Liquid-solid ratio)	B (Extraction temperature)	C (Methanol ratio)	D (Extraction time)	MAAs yield (mg/g)

1	0	1	1	0	135.24±3.82
2	0	-1	0	-1	123.26±3.14
3	0	0	1	-1	142.04±2.52
4	-1	1	0	0	135.73±1.38
5	-1	0	1	0	163.19±2.45
6	-1	0	-1	0	140.52±3.22
7	0	1	-1	0	136.42±2.79
8	0	0	-1	-1	128.45±4.11
9	0	0	-1	1	135.81±2.32
10	0	0	1	1	137.83±3.29
11	0	0	0	0	168.15±3.42
12	0	-1	0	1	150.81±0.97
13	1	1	0	0	101.04±1.43
14	0	0	0	0	178.60±3.52
15	1	0	-1	0	140.28±3.23
16	-1	0	0	1	165.44±2.91
17	0	0	0	0	169.20±3.14
18	0	1	0	-1	110.17±2.37
19	0	0	0	0	169.20±2.85
20	-1	0	0	-1	120.93±2.17
21	0	1	0	1	130.07±4.33
22	0	0	0	0	168.25±3.12
23	1	0	1	0	104.25±3.62
24	0	-1	-1	0	146.27±0.65
25	1	-1	0	0	146.72±3.26
26	1	0	0	1	118.25±2.27
27	1	0	0	-1	105.33±3.26
28	0	-1	1	0	135.27±3.41
29	-1	-1	0	0	154.13±2.96

Table S10 Significance tests and analysis of variance for the regression model of glyceroglycolipids extract from *E. kurome.*

Source	Sum of Squares	df	Mean Square	F-value	p-value
Model	11622.18	14	830.16	10.65	< 0.0001**
A-Liquid-solid ratio	2243.25	1	2243.25	28.79	< 0.0001**
B-Extraction temperature	968.22	1	968.22	12.43	0.0034**
C- Methanol ratio	8.22	1	8.22	0.1055	0.7502
D-Extraction time	972.54	1	972.54	12.48	0.0033**
AB	186.05	1	186.05	2.39	0.1446
AC	861.42	1	861.42	11.06	0.005**
AD	249.48	1	249.48	3.2	0.0952
BC	24.11	1	24.11	0.3094	0.5868
BD	14.63	1	14.63	0.1878	0.6714
CD	33.47	1	33.47	0.4295	0.5228
A^2	2469.14	1	2469.14	31.69	< 0.0001**
B^2	2183.07	1	2183.07	28.02	0.0001**
C^2	1145.76	1	1145.76	14.71	0.0018**
D^2	3412.88	1	3412.88	43.8	< 0.0001**
Residual	1090.82	14	77.92		
Lack of Fit	1011.5	10	101.15	5.1	0.0652
Pure Error	79.32	4	19.83		
Cor Total	12713	28			

Table S11 Response surface test design and results of glycolipids extraction from *U. lactuca*

Run	A (Liquid-solid ratio)	B (Extraction temperature)	C (Methanol ratio)	D (Extraction time)	MAAs yield (mg/g)
1	-1	0	0	1	143.23±1.23
2	0	1	0	1	146.80±4.36
3	1	1	0	0	161.11±3.35
4	0	0	0	0	217.36±2.72
5	0	0	0	0	226.13±1.84

6	0	0	1	1	147.13±4.53
7	-1	1	0	0	136.27±3.14
8	-1	0	-1	0	147.34±4.02
9	0	-1	0	-1	156.34±0.93
10	0	1	-1	0	137.18±3.14
11	0	0	-1	-1	153.26±4.56
12	0	-1	0	1	169.11±3.67
13	0	1	1	0	152.36±2.16
14	1	0	0	-1	166.42±3.22
15	0	0	0	0	221.43±3.93
16	1	0	-1	0	143.15±1.76
17	0	-1	-1	0	153.32±1.28
18	1	0	1	0	171.25±2.29
19	-1	0	1	0	153.47±2.00
20	-1	0	0	-1	160.11±1.93
21	0	-1	1	0	165.17±3.24
22	1	-1	0	0	185.33±3.51
23	0	1	0	-1	165.87±2.93
24	1	0	0	1	154.81±0.02
25	-1	-1	0	0	146.71±3.87
26	0	0	0	0	224.95±2.46
27	0	0	1	-1	165.80±1.24
28	0	0	0	0	218.14±3.41
29	0	0	-1	1	135.14±2.41

Table S12 Significance tests and analysis of variance for the regression model of glyceroglycolipids extract from *U. lactuca*.

Source	Sum of Squares	df	Mean Square	F-value	p-value
Model	21339.01	14	1524.21	31.19	< 0.0001**
A-Liquid-solid ratio	751.13	1	751.13	15.37	0.0015**

B-Extraction temperature	486.29	1	486.29	9.95	0.007**
C-Methanol ratio	613.33	1	613.33	12.55	0.0032**
D-Extraction time	426.97	1	426.97	8.74	0.0104*
AB	47.47	1	47.47	0.9714	0.3411
AC	120.67	1	120.67	2.47	0.1384
AD	6.94	1	6.94	0.1421	0.7119
BC	2.77	1	2.77	0.0567	0.8152
BD	253.45	1	253.45	5.19	0.039*
CD	0.0756	1	0.0756	0.0015	0.9692
A^2	6647.42	1	6647.42	136.02	< 0.0001**
B^2	6320.85	1	6320.85	129.34	< 0.0001**
C^2	9166.15	1	9166.15	187.56	< 0.0001**
D^2	6919.05	1	6919.05	141.58	< 0.0001**
Residual	684.18	14	48.87		
Lack of Fit	622.46	10	62.25	4.03	0.0955
Pure Error	61.72	4	15.43		
Cor Total	22023.19	28			

Table S13 Results and analysis of orthogonal experiment for process optimization of water retention agent preparation.

Runs	Levels				Water absorption ratio (g/g)
	Acrylic acid/ glyceroglycolipids extract	Initiator content	Cross-linking agent content	Neutralization degree of acrylic acid	
1	1	1	1	1	589.42
2	1	2	2	2	825.47
3	1	3	3	3	653.18
4	2	1	2	3	784.32
5	2	2	3	1	765.94
6	2	3	1	2	725.63
7	3	1	3	2	604.57
8	3	2	1	3	598.17
9	3	3	2	1	536.38
k1	689.36	989.16	637.74	630.58	$P<0.05$
k2	758.63	729.86	715.39	718.56	

k3	579.71	638.40	674.56	678.56
R	178.92	350.76	77.65	39.44
