# Supplementary figures and images for: Comprehensive Extraction and Biological Activities of Mycosporine-like Amino Acids and Glyceroglycolipids Extracts from Two Macroalgae Ecklonia kurome and Ulva lactuca
Source: Foods. 2025 Jan 29;14(3):440. doi: 10.3390/foods14030440 (PMC11817109; doi:10.3390/foods14030440)

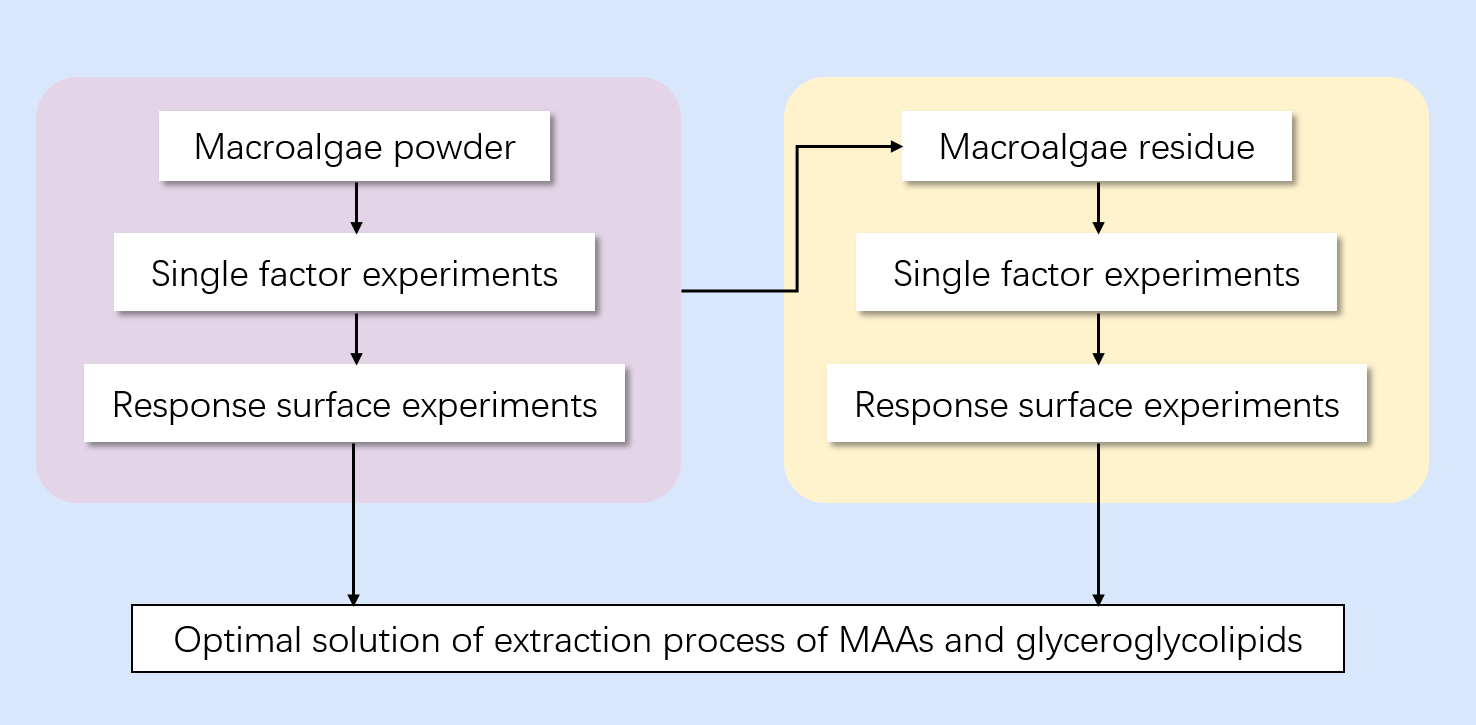

Supplement: Supplementary file 1 [file foods-14-00440-s001.zip › Figure S1.png]

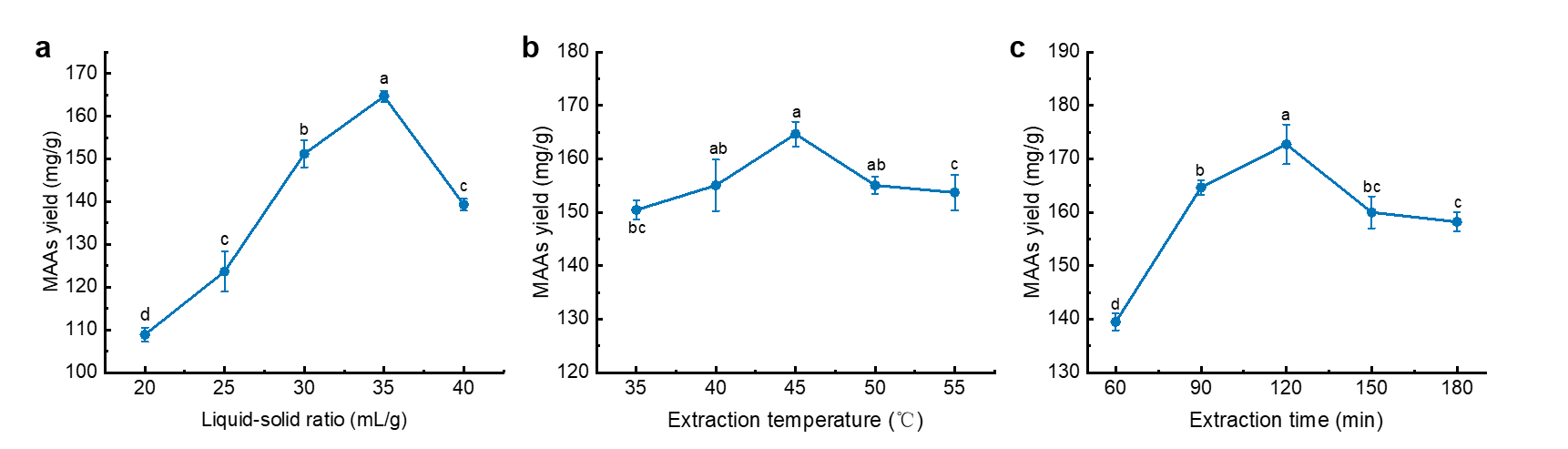

Supplement: Supplementary file 1 [file foods-14-00440-s001.zip › Figure S2.png]

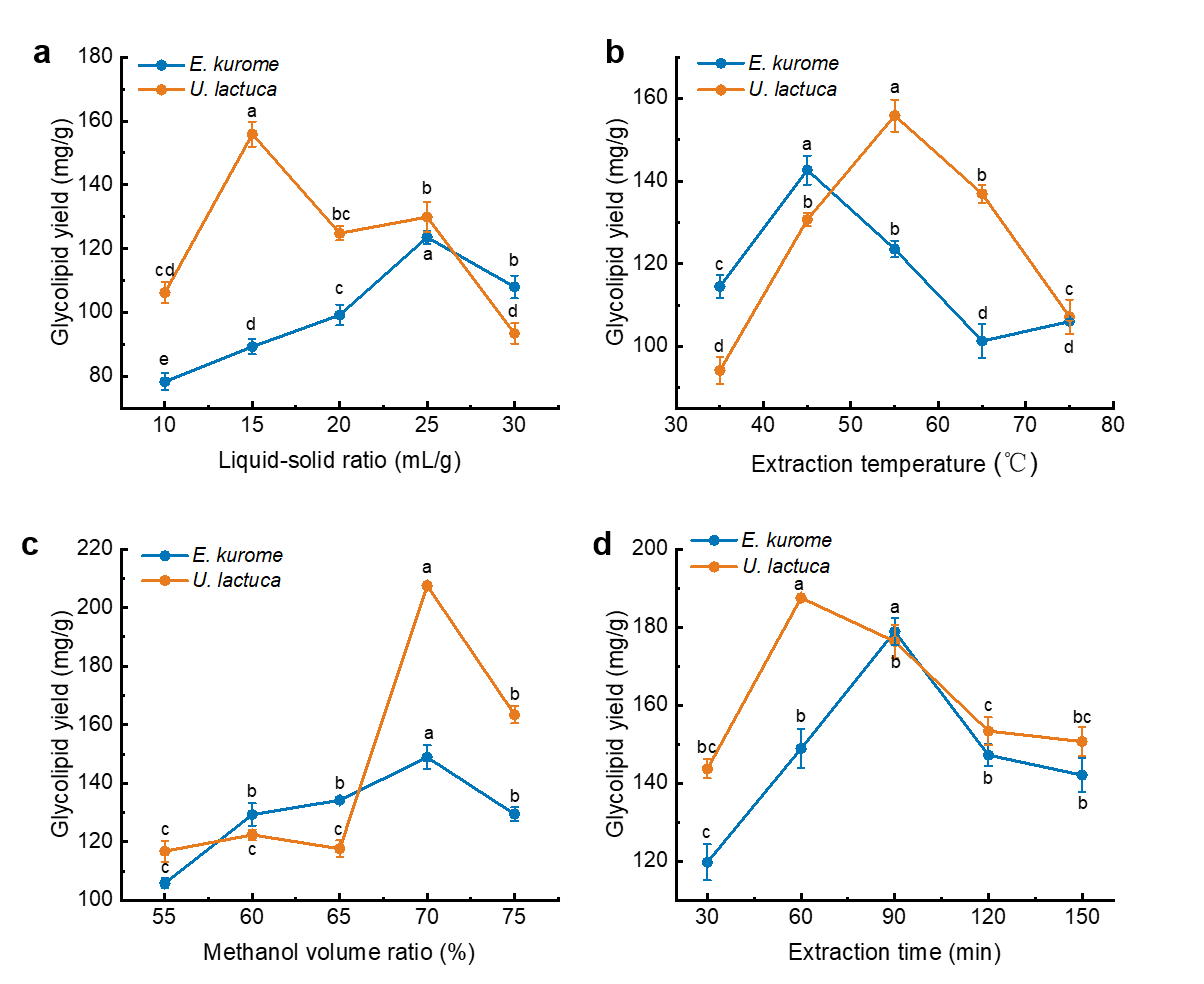

Supplement: Supplementary file 1 [file foods-14-00440-s001.zip › Figure S3.png]

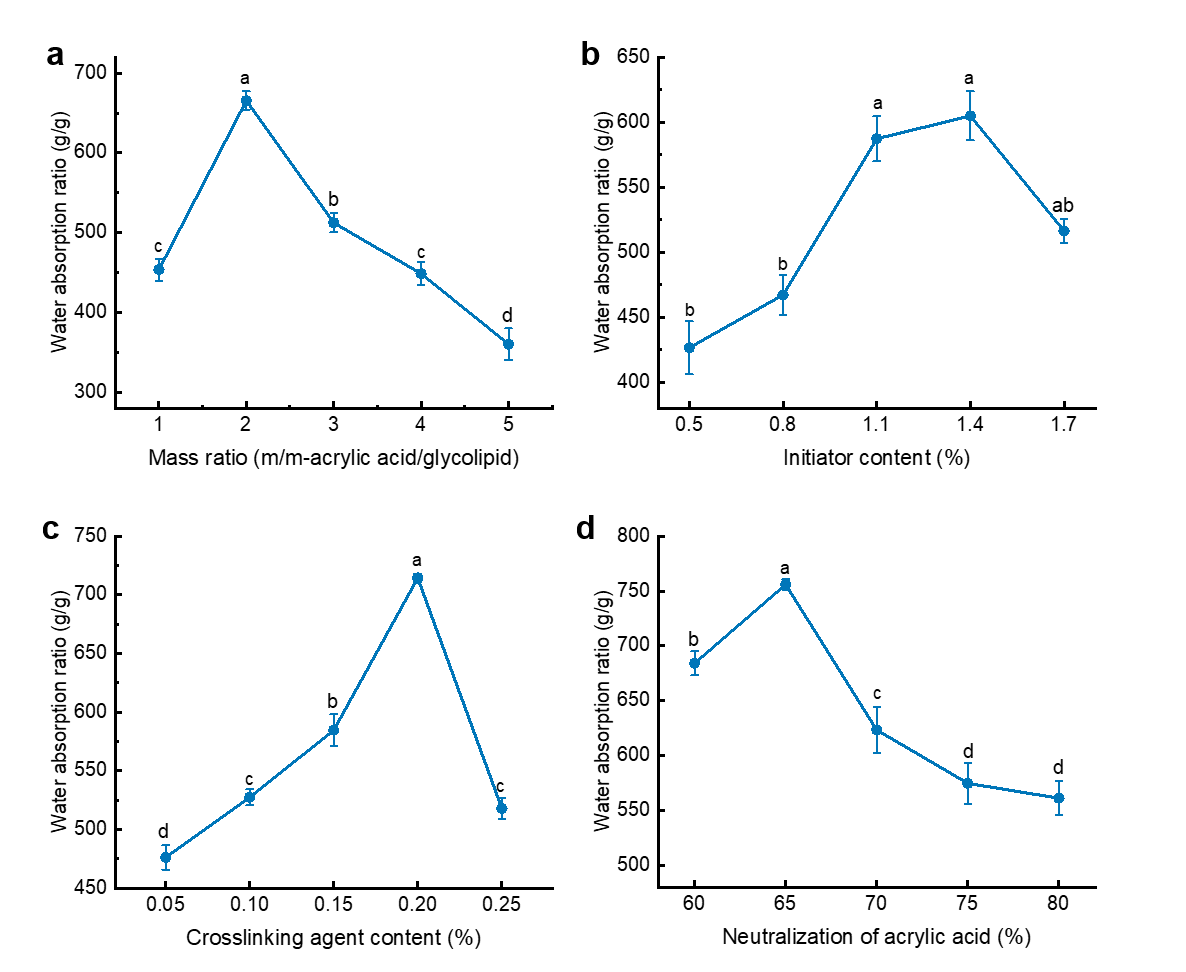

Supplement: Supplementary file 1 [file foods-14-00440-s001.zip › Figure S4.png]
